# Inter Individual Variations of the Fish Skin Microbiota: Host Genetics Basis of Mutualism?

**DOI:** 10.1371/journal.pone.0102649

**Published:** 2014-07-28

**Authors:** Sébastien Boutin, Christopher Sauvage, Louis Bernatchez, Céline Audet, Nicolas Derome

**Affiliations:** 1 Institut de Biologie Intégrative et des Systèmes (IBIS), Département de Biologie, Université Laval, Québec, Québec, Canada; 2 INRA, UR1052, Génétique et Amélioration des Fruits et Légumes (GAFL), Domaine St Maurice - Allée des Chênes, Montfavet, France; 3 Institut des sciences de la mer de Rimouski (ISMER), Université du Québec à Rimouski (UQAR), Rimouski, Québec, Canada; School of Environment & Life Sciences, University of Salford, United Kingdom

## Abstract

The commensal microbiota of fish skin is suspected to provide a protection against opportunist infections. The skin of fish harbors a complex and diverse microbiota that closely interacts with the surrounding water microbial communities. Up to now there is no clear evidence as to whether the host regulates the recruitment of environmental bacteria to build a specific skin microbiota. To address this question, we detected Quantitative Trait Loci (QTL) associated with the abundance of specific skin microbiota bacterial strains in brook charr (*Salvelinus fontinalis*), combining 16S RNA tagged-amplicon 454 pyrosequencing with genetic linkage analysis. Skin microbiota analysis revealed high inter-individual variation among 86 F2 fish progeny based upon the relative abundance of bacterial operational taxonomic units (OTUs). Out of those OTUs, the pathogenic strain *Flavobacterium psychrophilum* and the non-pathogenic strain *Methylobacterium rhodesianum* explained the majority of inter-individual distances. Furthermore, a strong negative correlation was found between *Flavobacterium* and *Methylobacterium*, suggesting a mutually competitive relationship. Finally, after considering a total of 266 markers, genetic linkage analysis highlighted three major QTL associated with the abundance of *Lysobacter*, *Rheinheimera* and *Methylobacterium*. All these three genera are known for their beneficial antibacterial activity. Overall, our results provide evidence that host genotype may regulate the abundance of specific genera among their surface microbiota. They also indicate that *Lysobacter, Rheinheimera* and *Methylobacterium* are potentially important genera in providing protection against pathogens.

## Introduction

The study of the beneficial effects of bacteria and their influence on host health is a growing field. Namely, research that has explored host microbiota variability in space and time suggests the presence of a host genetic component into the development of mutualism communities [1], [2]. Similar conclusions were found in several studies on twins and on their core gut microbiota [3], [4], [5], [6]. Furthermore, microbiome homeostasis seems to be the key to resistance against some diseases previously considered exclusively influenced by genetic factors [7]. To determine precisely which genes are involved in the recruitment of specific bacterial strains, some studies looked at gene expression in presence of symbiotic bacteria. Recent studies specifically targeted genes already known as being involved in innate or adaptive immunity (for example IgA) [8], [9], [10].

In fish, the influence of host genetic background on commensal bacterial community structure is poorly known. However, advances in the field of probiotic development indicate that endogenous bacteria are able to outcompete pathogens [11], [12]. Results obtained in aquaculture settings are consistent with previous work showing that non-pathogenic bacteria associated with animal mucosa can contribute to the host health by providing protection against pathogen infections [13], [14], [15], [16]. The genetic basis of the host immune response, especially at the major histocompatibility complex (MHC), toll-like receptor (TLR) and immunoglobulin loci, has been well studied [17], [18], [19], [20]. However, aside from the immune response, the influence of the host on the development of bacterial symbiosis remains poorly understood.

Here, the main objective of the present work was to investigate the potential link between host genotype and skin microbiota composition. As a host model, we focused on brook charr (*Salvelinus fontinalis*), a salmonid that harbors a complex and dynamic skin microbiota [21], [22]. The first specific objective sought to document the genetic variation present in the host population that might underpin the inter-individual variations in the skin microbiota. The second specific objective was to characterize the relationship (cooperation/competition) existing among the different bacterial Operational taxonomic units (OTU) within the skin mucus, which influences the overall structure of the skin microbial community. The third objective was to identify host genetic regions associated to the abundance of specific skin bacterial OTUs (i. e. quantitative trait loci (QTL) associated with the abundance of each constituent bacterial genus). Accordingly, we analyzed the taxonomic structure of skin bacterial community using 16S tagged-amplicon pyrosequencing in 86 F2 fish progenies obtained in the previous work of Sauvage *et al.* (2012). Inter-individual variation in abundance of each bacterial strain detected in host microbiota was afterwards projected on the results of the genetic linkage map in order to identify quantitative trait loci (QTL) of specific skin bacterial OTUs.

## Materials and Methods

### Ethics statement

All fish were reared and the experiment conducted strictly following guidelines required by the “Comité de Protection des Animaux de l'Université Laval (CPAUL, http://www.vrr.ulaval.ca/deontologie/cpa/index.html?accueil). The CPAUL reviewed and approved all experimental procedures used in this study.

### Fish sampling

The study population was generated using two different populations of brook charr. The first one (D) is a domestic population used in aquaculture for more than 100 years (Pisciculture de la Jacques Cartier - Cap-Santé, Québec, Canada). The other population (L), is an anadromous population from the Laval River near Forestville (North of the St. Lawrence River, Québec, Canada). Breeders from the L population were kept in captivity at the Station aquicole de l'ISMER (Québec, Canada, 48°31′N, 68°28′W) under natural photoperiod and temperature. Crosses between 10 sires of each population (L and D; F_0_ generation) with 10 dams (L and D) were performed to generate 10 full-sib outbred hybrid families (L×D - F_1_ generation). Subsequently, six F_1_ individuals were bi-parentally crossed to obtain three F_2_ families. The F_2_ family selected for the present study demonstrated the lowest post-hatch mortality rate (<2%) and the greatest abundance. Fish were raised in the same tank and had fasted for 12 hours before sampling. Fish were measured (mean  = 28.8±1.77 cm) and weighed (mean  = 276.7±59.48 g) to calculate Fulton index [23].

Mucus was sampled using a sterile swab on the same area for all the fish [24]. We choose to sample the skin between the adipose fin and the caudal fin because this area was undisturbed by fish handling. Samples were stored in a sterile micro-centrifuge tube containing lysis buffer (Tris 50 mM, EDTA 40 mM, sucrose 0.75 g) and immediately stored in −80°C until DNA extraction.

### DNA extraction

DNA was extracted using a modified protocol of salt-extraction from Aljanabi & Martinez (1997). During the first lysis step, 22.6 µL of lysozyme (40 mg/mL) was added to the sample and incubated 45 minutes at 37°C. After this step, 22.6 µL of proteinase K (20 mg/µL) and 90 µL of SDS (10%) were added to the lysate and incubated at 55°C over night with agitation. The aqueous phase was transferred into a clean Eppendorf tube containing 600 µL of NaCl 6M, mixed and centrifuged 20 min at 14,800 rpm. The supernatant was transferred again into a clean Eppendorf tube containing 1 volume of cold isopropanol, mixed and stored 30 minutes at −20°C. The mixture was centrifuged 20 minutes at 14,800 rpm and the supernatant was thrown away. The pellet was washed with cold ethanol 70%, air-dried and finally resuspended in 25 µL of sterile MilliQ H_2_O. Subsequently, DNA integrity and quantity were controlled using a Nanodrop instrument (ND-1000, Nanodrop).

### Microbial 16S pyrosequencing

Each DNA sample, the 16S gene was PCR amplified using Takara Ex taq premix (Fisher). All PCR reactions were performed in a reaction volume of 50 µL containing 25 µL of premix Taq, 1 µM of each primer and sterile MilliQ H_2_O to up to 50 µL. A general reverse primer (R519) combined with B primer (Roche) was used for amplifications in combination with one of 86 uniquely tagged forward primers (F63-targeted) combined with A primer (Roche) (for primer sequences see [25], [26]. The mean length of the amplified fragment was 450 bp. This procedure facilitates the parallel sequencing of thousands of samples on the same run and to reassign each reads to their respective samples. PCR conditions were applied as follows: after a denaturing step of 30 s at 98°C, samples were processed through 30 cycles consisting of 10 sec at 98°C, 30 sec at 55°C, and 30 sec at 72°C. The final extension step was done at 72°C for 4 min 30 sec. Following the amplification step, samples were purified using AMPure Beads (Beckman Coulter Genomics). Samples were adjusted to 100 µL with EB (Qiagen), 63 µL of beads were added. Samples were mixed and incubated for 5 min at RT. Using a Magnetic Particle Concentrator (MPC), the beads were pelleted against the wall of the tube and supernatant was removed. The beads were washed twice with 500 µL of 70% ethanol and incubated for 30 sec each time. Supernatant was removed and beads were air dried for 5 min. Tubes were removed from the MPC and 24 µL of EB were added. Samples were vortexed in order to suspend the beads. Finally, using the MPC, the beads were pelleted against the wall once more and supernatant was transferred to a new clean tube. Samples were quantified with Nanodrop before the amplification step. Amplicons were then quantified with Quant-iT PicoGreen dsDNA Assay Kit and mixed equally before being sent to the Plateforme d'Analyses Biomoléculaires (Institut de Biologie Intégrative et des Systèmes, Université Laval) for pyrosequencing on a 454 GS-FLX DNA Sequencer with the Titanium Chemistry (Roche), according to manufacturer's procedure.

### 16S sequence analysis

All sequences are available on MG-RAST server (MG-RAST IDs: 4539915.3). The data were analyzed in two steps. First, CLC Genomics Workbench 3.1 (CLC Bio, Aarhus, Denmark CLC workbench BIO) was used to trim sequences for quality, recover and remove the primers' sequences and tags (minimum average quality score: 35 for a window of 50, number of differences to the primer sequence  = 0, maximum number of differences to the barcode sequence  = 0, number of ambiguous base calls  = 0, maximum homopolymer length  = 8). In a second step, pre-processing and analysis were performed using the MOTHUR software [27]. All datasets were checked for chimeras with the chimera slayer algorithm implemented in MOTHUR. Standardization of the different samples was done by using the zscore which calculates the normalized abundance as follows: normalized abundance  =  (abundance - mean) / standard deviation [3], [28], [29]. An alternative method for normalization was also tested, which consisted of subsampling equal number of reads from each sample [30]. This method greatly reduced the numbers of sequences (224 sequences per samples), but gave the same results as zscore (Figure S2, Figure S3, Table S1). Therefore we preferred to keep zscore normalization to base our conclusions on a larger dataset. We used the Operational Taxonomic Unit-based method described by Costello *et al.* (2009) because it is not biased towards a predefined taxonomy. One index was retained to assess the quality of pyrosequencing: the sequence coverage index (Good's estimator). The sequence coverage index is a metric used to estimate the quality of the depth sequencing. All sequences were clustered into Operational Taxonomic Unit (OTU) using a 97% identity threshold and OTU were classified from phylum to genus using the program MOTHUR with the default settings. For all interesting OTUs (explaining the inter-individual variation or linked to a genetic region), we extracted all the sequences classified in that OTU and used the single best BLAST hit to identify the different species. Statistical differences in the abundance of each OTU were calculated with the software for metagenomic analysis (Metastats).

To visualize potential differences across host progenies in terms of mucus bacterial community structure, distances between those communities were computed using the Yue & Clayton measure of dissimilarity (Thetayc). This index developed by Yue and Clayton (2005) is a measure of dissimilarity between the structures of two communities, meaning that this calculator takes in account the abundances of each OTU. Then, Distances were represented using a dendrograms based on this index and statistical robustness of the dendrogram was determined by a Unifrac weighted test. This test allows determining whether any of the samples have a significantly different structure than the other samples. A random (Monte Carlo) permutation test was performed to test whether or not the distance between two communities was greater than expected by chance alone.

To visualize the distances between communities, we also applied a Principal Coordinate Analysis (PCoA) based on the Yue & Clayton measure of dissimilarity (Thetayc) using an eigenvector-based approach to represent multidimensional data in as few dimensions as possible. This method allows determining which OTUs are responsible for shifting the samples along the PCoA axes by measuring the correlation of the relative abundance of each OTU clustered in genera with the axes of the PCoA dataset. Statistical differences in the abundance of each OTU was calculated to highlight which genera were the causative agents of the differentiation between groups obtained in the dendrogram based upon the TethaYC index [31]. All statistical analyses and graphics were carried out in the R environment (http://www.r-project.org).

### QTL detection

QTL analysis was carried out using the [R] package J/qtl [v. 1.3.1, August 2012, http://churchill.jax.org/software/jqtl.shtml/]. QTL were projected on the consensus brook charr linkage map (see Sauvage *et al.*, 2012) according to the following steps. (1) A single QTL analysis was performed using the Haley-Knott (HK) regression method (10,000 permutations) to reveal which LGs were carrying QTL. The most probable position of the QTL was defined at the position giving the largest logarithm of odds (LOD) score indicating the QTL was fixed. (2) A two QTL model based upon the Haley-Knott regression was used to refine the QTL detection across the genome with a resolution of 5 cM and eventually to detect two QTL on a single LG linked to a particular trait. (3) The best model fitting our data was used to compute the percent variance explained (PVE expressed in %) by the QTL. The chromosome-wide and the genome-wide thresholds were calculated for each LG using 10,000 permutations. The 1.5 LOD confidence intervals were determined for all analyses following the Bayesian method implemented in the “bayesint” function in R/qtl. The bayesint function calculates an approximate interval (end points around the maximum LOD) for a given chromosome using the genome scan output.

## Results

Mucus samples were obtained from 86 F2 fish progenies (44 males and 42 females) issued from the same family. A total of 87,940 high-quality, classifiable sequences were obtained with an average number of 1022±540 per sample, which were subsequently classified in OTUs [1]. All of these sequences were successfully clustered into 9520 OTUs with 97% identity. A final trimming step was performed to focus on the most abundant OTU (which are represented by at least 10 sequences for the whole project) and result in a dataset containing 71,719 sequences (81% of the initial data set) clustered in 192 OTUs. The depth of sequencing and the coverage were estimated using the Good's estimator index; the coverage found was always higher than 90% except for one sample (#127), which exhibited 76% of coverage (Table 1, Figure S1). The number of OTUs per sample was not equally distributed and the mean number was 156.5±81.7 (ranging from 33 to 368). The alpha-diversity was estimated by calculating the non-parametric index of Shannon (npShannon) because of the non-equally distributed abundance of each OTU in the samples. The npShannon index ranged from 0.29 to 3.45, with a mean index of 1.43±0.83.

**Table 1:** Descriptive statistics of sequencing to determine the microbiotoa for each individual.

Sample ID	number of reads	coverage	npshannon index	Number of OTU total	Number of OTU >10 reads
**92**	229	0.955975	1.690045	76	19
**93**	1584	0.99131	0.436879	74	28
**94**	669	0.977401	1.507996	242	20
**95**	738	0.986166	1.416086	226	27
**96**	503	0.953079	1.395581	149	29
**97**	385	0.946203	1.774329	86	33
**98**	955	0.98389	0.663968	79	30
**99**	770	0.978852	1.306709	114	29
**100**	1018	0.985946	0.658787	107	30
**101**	1482	0.994792	0.842304	188	21
**102**	1667	0.992283	0.691189	96	31
**103**	711	0.99179	0.946539	97	23
**104**	1072	0.987599	0.719994	159	25
**105**	1662	0.991645	0.371924	73	26
**106**	1304	0.995734	0.658557	93	18
**107**	1617	0.99416	0.290016	49	21
**108**	1237	0.990451	0.634742	73	30
**109**	1063	0.987778	0.679124	137	26
**110**	752	0.97281	0.656197	105	29
**111**	731	0.986217	0.63276	79	22
**112**	628	0.975779	0.630137	63	23
**113**	892	0.983549	0.446389	49	24
**114**	1333	0.993127	0.713271	127	22
**115**	1109	0.983313	1.020009	266	35
**116**	532	0.967611	2.709733	269	32
**117**	649	0.963145	2.108945	237	41
**118**	404	0.923977	2.645631	222	29
**119**	969	0.97625	1.019139	177	40
**120**	581	0.967647	1.975631	205	26
**121**	468	0.948413	2.692236	202	34
**122**	744	0.980551	0.876389	117	27
**123**	953	0.983204	0.832745	133	32
**124**	530	0.967846	2.29117	186	30
**125**	797	0.977901	0.703269	79	30
**126**	411	0.97561	0.904985	116	18
**127**	242	0.76	3.452235	182	32
**128**	1230	0.989637	0.504536	69	26
**129**	831	0.978723	1.537117	115	41
**130**	1449	0.983421	0.548428	123	38
**131**	499	0.969697	2.118408	144	29
**132**	431	0.945813	2.133064	181	25
**133**	494	0.96114	1.408929	117	35
**134**	746	0.980254	0.508333	38	22
**135**	662	0.977887	2.41624	180	36
**136**	754	0.987755	0.956343	118	18
**137**	2008	0.993154	0.557917	74	30
**138**	714	0.976619	1.632525	101	36
**139**	798	0.967632	1.534968	129	35
**140**	469	0.917197	3.267019	261	35
**141**	224	0.980892	0.662835	33	6
**142**	517	0.957377	2.388226	180	36
**143**	483	0.947195	2.446711	157	36
**144**	414	0.958848	3.124298	188	38
**145**	930	0.975443	1.391363	168	43
**146**	769	0.979661	2.024708	140	28
**147**	1219	0.995122	1.641489	118	17
**148**	1254	0.98244	1.127563	148	40
**149**	1074	0.972678	2.309049	318	55
**150**	1108	0.980583	2.793443	321	47
**151**	3082	0.998643	0.332436	58	16
**152**	1429	0.996561	1.820656	203	25
**153**	1955	0.995286	0.815557	167	27
**154**	1449	0.983366	1.74373	354	54
**155**	1074	0.973611	1.716763	321	41
**156**	1016	0.989316	0.588719	62	21
**157**	1483	0.984836	1.374063	216	46
**158**	2004	0.998944	0.791157	44	10
**159**	2043	0.997359	0.812388	92	18
**160**	2468	0.995741	0.305525	70	23
**161**	1215	0.979566	1.928985	346	48
**162**	1904	0.995286	0.821609	164	37
**163**	1046	0.985799	1.569251	188	40
**164**	1145	0.993814	0.744587	98	19
**165**	995	0.981481	1.421104	243	37
**166**	725	0.97921	2.459141	211	36
**167**	727	0.988743	2.142562	131	24
**168**	841	0.98908	2.297402	151	31
**169**	552	0.968116	2.76388	178	35
**170**	1204	0.99177	1.912832	162	32
**172**	2010	0.993946	0.610072	155	36
**173**	1454	0.987296	1.893132	316	51
**174**	1056	0.979651	2.949199	335	53
**175**	824	0.95098	2.845177	368	46
**176**	851	0.96729	2.039242	202	47
**177**	778	0.985507	2.23077	221	25
**178**	2142	0.998093	0.359619	52	13

The coverage was estimated by a Good's estimator index. npshannon: non parametric index of Shannon. OTU classification was done with a treshold of 97% identity.

**Figure 1:**
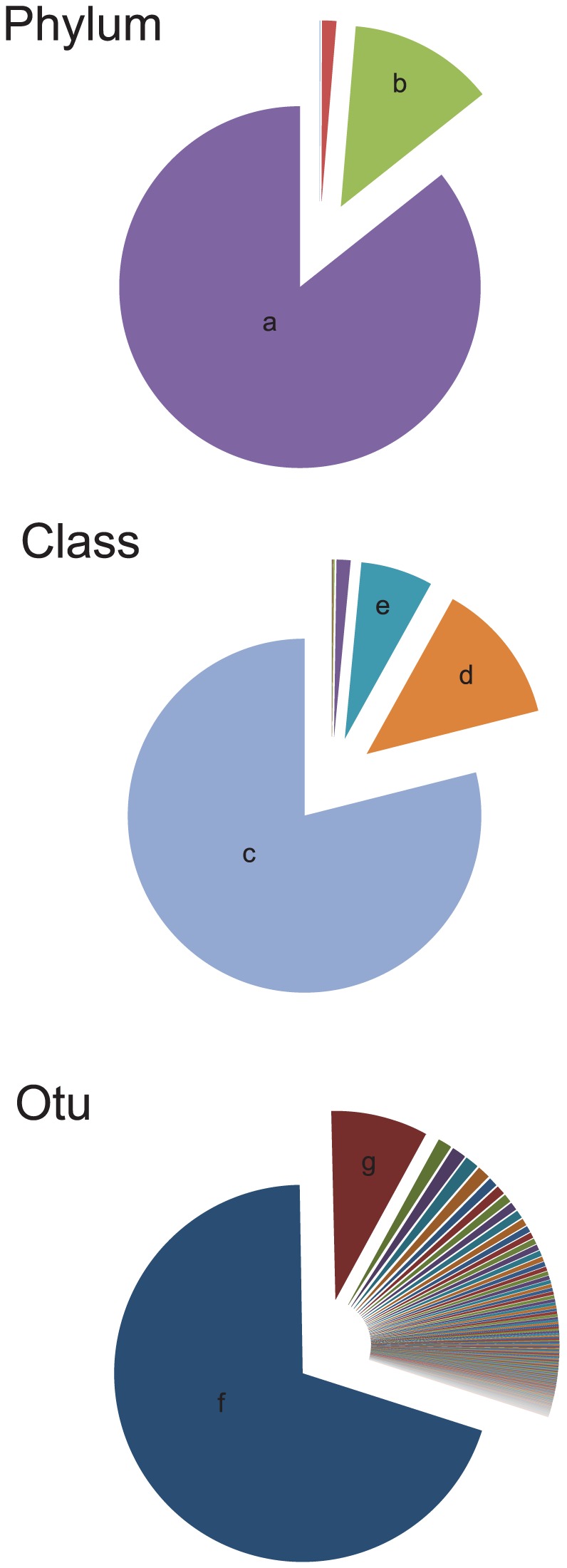
Taxonomic structure of the bacterial community of fish skin mucus at three different taxonomic levels: Phylum, Classl and OTU. a) Proteobacteria; b) Bacteroidetes, c) Alphaproteobacteria, d) Flavobacteria, e) Gammaproteobacteria, f) OTU 50 (*Methylobacterium rhodesianum*), g) OTU 36 (*Flavobacterium psychrophilum*).

Phylum, class, and OTU composition of all samples were represented in the Figure 1. Most of the OTUs belong to the *Proteobacteria* phylum (88.7%) and the *Bacteroidetes* phyla (9.7%). At the class level, most of the OTUs were classified as *Alpha-proteobacteria* (78.9%), *Gamma-proteobacteria* (6.5%) and *Flavobacteria* (13%). At the OTU level, the most abundant sequence was identified via BLAST as *M. rhodesianum* (69.8%) followed by *F. psychrophilum* (8%) (Figure 1 and Table S1).

Male and female brook charr harbored slightly different microbial communities. The Unifrac weighted distance between samples from males and females was statistically significant indicating that structures of bacterial communities were differentiated according to the sex of the host (Unifrac Score: 0.241695; p-value <0.001). However, there was not a sex difference in the two most abundant species, as this difference was based on 22 OTUs classified on 20 genera and 21 species, all of them being less abundant than 0.1% (Table 2).

**Table 2:** Taxonomic variation observed between male and female.

Condition	N° OTU	Best Hit on Blast	Identity	Relative abundance
**More abundant in female**	82	*Amaricoccus kaplicensis*	98%	0.00037647
	115	*Loktanella salsilacus*	99%	0.00015338
	6	*Methylovirgula ligni*	91%	0.00026492
	163	*Micrococcus antarcticus*	99%	0.00061351
	88	*Paracoccus yeei*	94%	0.00019521
	75	*Pseudochrobactrum kiredjianiae*	99%	0.00050196
	128	*Psychrobacter faecalis*	99%	0.00100392
	131	*Rheinheimera pacifica*	99%	0.00057168
	7, 11	*Singularimonas variicoloris*	89%	0.00037647
	104	*Sphingopyxis witflariensis*	99%	0.00015338
	113	*Stella humosa*	100%	0.00018126
	142	*Stenotrophomonas nitritireducens*	99%	0.00022309
**More abundant in male**	186	*Flavobacterium aquatile*	97%	0.00029281
	190	*Hyphomicrobium sulfonivorans*	95%	0.00039041
	4	*Methylosinus sporium*	92%	0.00027887
	176	*Polynucleobacter necessarius subsp. Asymbioticus*	99%	0.00015338
	129	*Pseudomonas hibiscicola*	99%	0.00075294
	12	*Pseudorhodoferax soli*	85%	0.00018126
	140	*Psychrobacter alimentarius*	99%	0.00015338
	116	*Rhodobacter blasticus*	95%	0.00016732
	175	*Simplicispira limi*	99%	0.00034858

This table summarize the OTUs differentially abundant between skin mucus communities between male and female (Metastat using a FDR correction, p-value<0.01).

**Figure 2:**
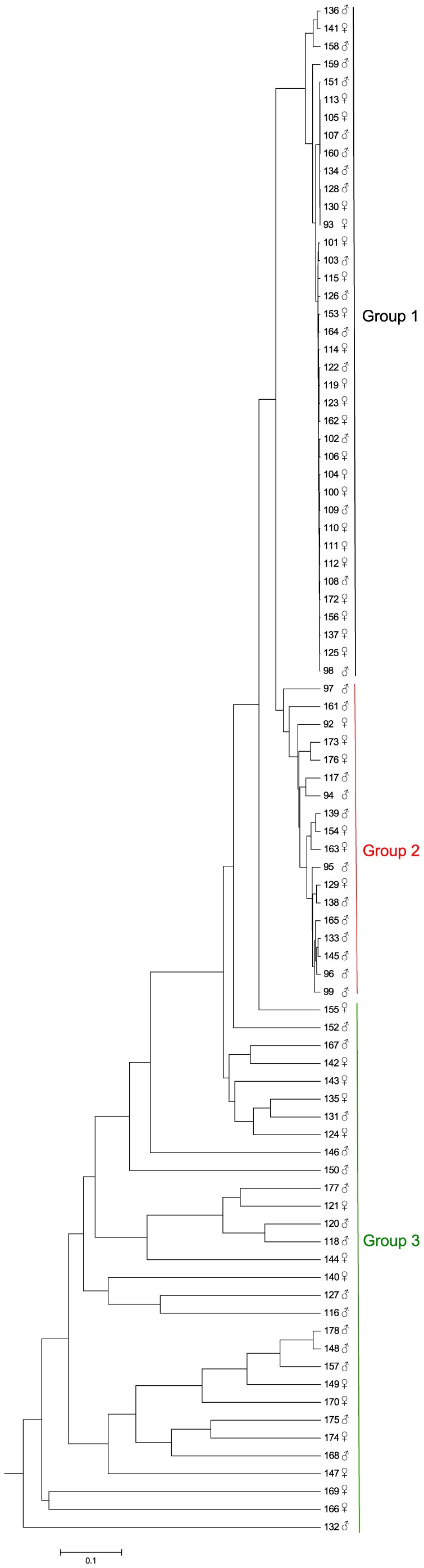
Dendrogram analysis based upon ThetaYC index of bacteria found on the skin of the 86 brook charr individuals. Groups are defined with the Weighted Unifrac distance. The first and second groups are composed of closely related bacterial communities. The third group is an assemblage of dissimilar communities and is considered as an outgroup. The most differentiated groups are groups 1 and 3 (Unifrac Score: 0.710811, p<0.0010) followed by the distance between groups 2 and 3 (Unifrac Score: 0.685361, p<0.0010), and the distance between groups 1 and 2 (Unifrac Score: 0.401674, p<0.0010).

**Figure 3:**
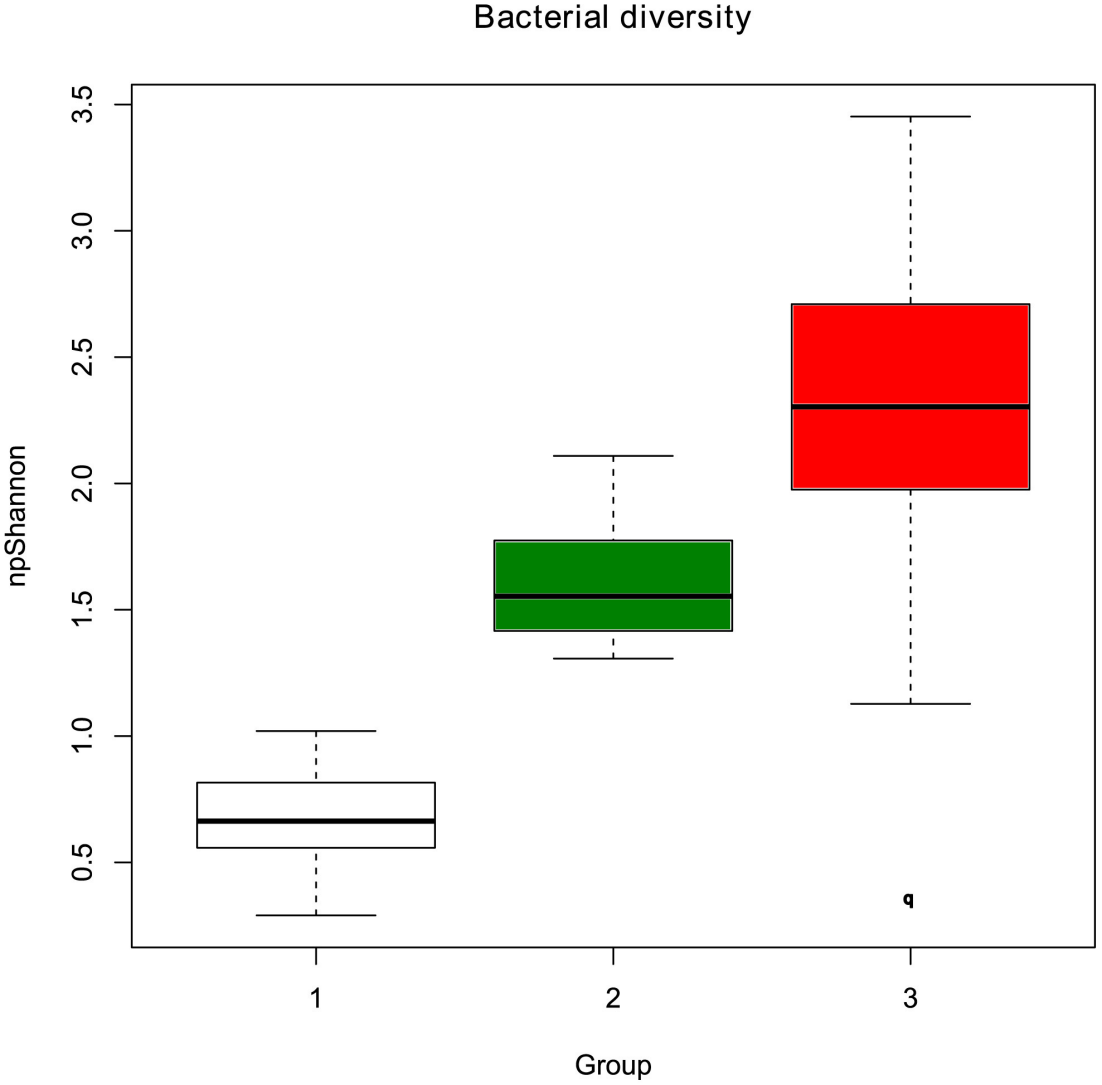
Alpha diversity of each group. Each group was defined by the dendrogram analysis based upon ThetaYC index (see figure 2). Alpha-diversity was calculated by the non-parametric index of Shannon.

**Figure 4:**
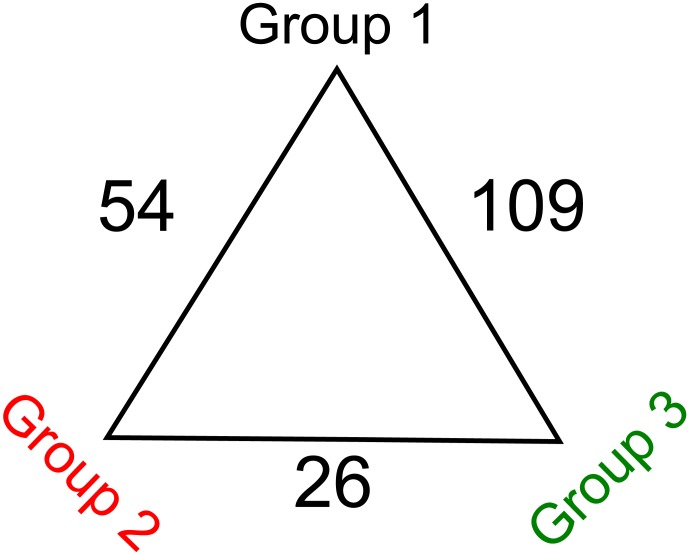
Descriptive analysis of the Metastats. Numbers indicates the numbers of OTUs differentially present in the different groups of individuals. For details on the identities of the OTUs, please refer to the table 3; 4 and 5.

In terms of community structure, clustering based on ThetaYC index clearly defined two groups of samples, whilst a third group was less well defined and composed of highly variable communities (Figure 2). There was little variation among samples in the first and second group (group 1 and group 2). The third group (group 3) was an assemblage of dissimilar communities and is considered as an external group. The variation of the alpha-diversity visualized by the npShannon index (Figure 3) indicated that alpha-diversity increases from group 1 to group 3. Furthermore, the bacterial communities within the third group were more diverse than groups 1 and 2. The same clustering was found with the normalization by subsampling (Figure S2, Figure S3, table S1). The difference between each group was greater than the difference between males and females (Figure 2). The most differentiated groups were groups 1 and 3 (Unifrac Score: 0.710811, p-value <0.001) followed by the distance between groups 2 and 3 (Unifrac Score: 0.685361, p-value <0.001), and the distance between groups 1 and 2 (Unifrac Score: 0.401674, p-value <0.001). This was correlated with the numbers of OTU that were differentially abundant between those 3 groups (Figure 4). The divergence between groups 1 and 2 was based upon 54 OTU classified in 26 genera and 41 species (Table 3). The microbiota of the first group was dominated by *M. rhodesianum*, and host individuals from this group were tightly clustered. The second group was more diverse, dominated by *Flavobacterium* and had a lower abundance of *M. rhodesianum.* For groups 1 and 3, the difference was based upon 109 OTU classified in 45 genera and 70 species (Table 4). Finally, 26 OTU were differently abundant between group 2 and 3 and classified in 17 genera and 22 species (Table 5).

**Table 3:** Detailed information related to the OTU differentially abundant between individuals belonging to group 1 and group 2.

N° OTU	mean abundance in group 1	mean abundance in group 2	Best hit on Blast
**36**	0.00484	0.037926	*Flavobacterium psychrophilum*
**122**	0.000778	0.010066	*Pseudomonas cedrina subsp. Fulgida*
**37**	0.000471	0.006593	*Flavobacterium terrigena*
**68**	0.0014	0.006629	*Methylocella palustris*
**128**	0	0.004734	*Psychrobacter faecalis*
**69**	0.000531	0.004942	*Pseudorhodobacter ferrugineus*
**190**	0	0.004394	*Hyphomicrobium sulfonivorans*
**139**	0.000153	0.003833	*Lysobacter capsici*
**70**	0.000831	0.0043	*Methylobacterium salsuginis*
**29**	0.000028	0.003344	*Methylobacterium adhaesivum*
**67**	0.000518	0.003772	*Rhodobacter blasticus*
**127**	0.000218	0.003382	*Acinetobacter haemolyticus*
**21**	0.000117	0.002977	*Flavobacterium aquatile*
**74**	0	0.002743	*Paracoccus yeei*
**52**	0.000331	0.002911	*Sphingopyxis alaskensis*
**126**	0.000969	0.003524	*Pseudomonas xanthomarina*
**89**	0	0.002528	*Sphingomonas dokdonensis*
**27**	0.000113	0.001814	*Sphingopyxis chilensis*
**63**	0.00071	0.002374	*Paracoccus haeundaensis*
**141**	0.000156	0.001642	*Acinetobacter johnsonii*
**84**	0.000079	0.001484	*Pseudorhodobacter ferrugineus*
**38**	0.000063	0.001334	*Sejongia jeonii*
**138**	0.000014	0.001205	*Acinetobacter junii*
**192**	0	0.001099	*Methylovirgula ligni*
**81**	0.000248	0.00132	*Ruegeria atlantica*
**8**	0	0.001062	*Methylosinus sporium*
**6**	0	0.001033	*Methylovirgula ligni*
**12**	0	0.001029	*Pseudorhodoferax soli*
**86**	0.000282	0.00131	*Methylobacterium rhodesianum*
**32**	0.000125	0.001139	*Methylobacterium zatmanii*
**22**	0.000011	0.000871	*Flavobacterium psychrophilum*
**107**	0.000082	0.000932	*Rhodobacter capsulatus*
**43**	0.000258	0.001098	*Flavobacterium aquatile*
**94**	0.000073	0.000886	*Rhodobacter sphaeroides*
**189**	0.000043	0.000809	*Methylomicrobium album*
**78**	0.000047	0.000761	*Rhodobacter capsulatus*
**91**	0.000226	0.000929	*Caulobacter leidyia*
**46**	0.00002	0.000712	*Flavobacterium psychrolimnae*
**101**	0.00042	0.001111	*Brevundimonas variabilis*
**154**	0.00005	0.000728	*Methylobacterium adhaesivum*
**82**	0.000156	0.000815	*Amaricoccus kaplicensis*
**54**	0.000176	0.000782	*Methylopila capsulata*
**19**	0.000154	0.000756	*Brevundimonas variabilis*
**71**	0	0.00054	*Sphingomonas faeni*
**182**	0.000046	0.000579	*Flavobacterium aquatile*
**90**	0.000282	0.000812	*Methylobacterium adhaesivum*
**44**	0.000094	0.000609	*Flavobacterium psychrophilum*
**143**	0	0.000513	*Dokdonella koreensis*
**112**	0	0.000511	*Sphingomonas sanguinis*
**97**	0	0.00049	*Hyphomicrobium facile subsp. Tolerans*
**105**	0	0.000359	*Paracoccus haeundaensis*
**137**	0	0.000339	*Acinetobacter johnsonii*
**75**	0.000558	0	*Pseudochrobactrum kiredjianiae*
**50**	0.889343	0.677999	*Methylobacterium rhodesianum*

Differentially abundancy were calculated by using Metastats (using a FDR correction, p<0.01).

**Table 4:** Detailed information related to the OTU differentially abundant between individuals belonging to group 1 and group 3.

OTU	mean abundance in group 1	mean abundance in group 3	Best hit on Blast
**36**	0.00484	0.15627	*Flavobacterium psychrophilum*
**52**	0.000331	0.052599	*Sphingopyxis alaskensis*
**51**	0.002582	0.045797	*Sphingomonas paucimobilis*
**119**	0.001237	0.034255	*Pseudomonas peli*
**125**	0.001567	0.030681	*Acinetobacter lwoffii*
**120**	0.002302	0.030821	*Rheinheimera texasensis*
**63**	0.00071	0.027857	*Paracoccus haeundaensis*
**122**	0.000778	0.018741	*Pseudomonas cedrina subsp. Fulgida*
**61**	0.003372	0.02093	*Porphyrobacter dokdonensis*
**21**	0.000117	0.011826	*Flavobacterium aquatile*
**127**	0.000218	0.00999	*Acinetobacter haemolyticus*
**55**	0	0.008105	*Bradyrhizobium pachyrhizi*
**54**	0.000176	0.007992	*Methylopila capsulata*
**162**	0.002112	0.009611	*Knoellia sinensis*
**126**	0.000969	0.008421	*Pseudomonas xanthomarina*
**67**	0.000518	0.007951	*Rhodobacter blasticus*
**69**	0.000531	0.007849	*Pseudorhodobacter ferrugineus*
**81**	0.000248	0.006693	*Ruegeria atlantica*
**134**	0.000245	0.00635	*Acinetobacter lwoffii*
**143**	0	0.005914	*Dokdonella koreensis*
**129**	0	0.005644	*Pseudomonas hibiscicola*
**124**	0	0.004908	*Nevskia soli*
**40**	0.000014	0.004576	*Flavobacterium aquatile*
**38**	0.000063	0.004547	*Sejongia jeonii*
**68**	0.0014	0.005784	*Methylocella palustris*
**71**	0	0.004232	*Sphingomonas faeni*
**79**	0.000259	0.004408	*Rhodobacter capsulatus*
**78**	0.000047	0.003657	*Rhodobacter capsulatus*
**37**	0.000471	0.004035	*Flavobacterium terrigena*
**141**	0.000156	0.003673	*Acinetobacter johnsonii*
**156**	0.000033	0.003487	*Sphingopyxis alaskensis*
**39**	0	0.00318	*Flavobacterium psychrophilum*
**24**	0.000095	0.00295	*Pseudomonas peli*
**106**	0.000106	0.002945	*Bosea vestrisii*
**137**	0	0.002615	*Acinetobacter johnsonii*
**101**	0.00042	0.002997	*Brevundimonas variabilis*
**97**	0	0.002498	*Hyphomicrobium facile subsp. Tolerans*
**139**	0.000153	0.002639	*Lysobacter capsici*
**157**	0.00006	0.002488	*Sphingomonas sanguinis*
**116**	0	0.002339	*Rhodobacter blasticus*
**177**	0.000289	0.00255	*Aquabacterium commune*
**25**	0.000069	0.002236	*Acinetobacter lwoffii*
**182**	0.000046	0.002172	*Flavobacterium aquatile*
**47**	0	0.002082	*Flavobacterium psychrophilum*
**84**	0.000079	0.00214	*Pseudorhodobacter ferrugineus*
**164**	0.000219	0.002164	*Rhodococcus fascians*
**105**	0	0.001788	*Paracoccus haeundaensis*
**43**	0.000258	0.002022	*Flavobacterium aquatile*
**180**	0.000017	0.001709	*Acidovorax defluvii*
**92**	0.000147	0.001809	*Sphingopyxis witflariensis*
**4**	0	0.00161	*Methylosinus sporium*
**145**	0	0.001417	*Acinetobacter johnsonii*
**5**	0	0.001415	*Magnetospirillum magnetotacticum*
**88**	0.000036	0.00143	*Amaricoccus veronensis*
**3**	0	0.001358	*Rhodobacter changlensis*
**138**	0.000014	0.001365	*Acinetobacter junii*
**70**	0.000831	0.002052	*Methylobacterium salsuginis*
**184**	0	0.001156	*Flavobacterium aquatile*
**22**	0.000011	0.001144	*Flavobacterium psychrophilum*
**112**	0	0.001106	*Sphingomonas sanguinis*
**118**	0.000062	0.001143	*Haematobacter missouriensis*
**93**	0.000029	0.001093	*Roseomonas mucosa*
**152**	0.000028	0.001009	*Rhodobacter blasticus*
**35**	0.00005	0.000936	*Sphingomonas sanguinis*
**148**	0.000033	0.000915	*Psychrobacter arcticus*
**172**	0	0.000864	*Sphingomonas sanguinis*
**146**	0	0.000832	*Pseudomonas mohnii*
**94**	0.000073	0.000865	*Rhodobacter sphaeroides*
**140**	0	0.000775	*Psychrobacter alimentarius*
**160**	0.000061	0.000823	*Sphingomonas faeni*
**83**	0.000128	0.000877	*Paracoccus aminovorans*
**42**	0.000323	0.001071	*Flavobacterium psychrophilum*
**186**	0	0.000711	*Flavobacterium aquatile*
**44**	0.000094	0.000796	*Flavobacterium psychrophilum*
**117**	0.000029	0.000731	*Sphingopyxis taejonensis*
**107**	0.000082	0.000766	*Rhodobacter capsulatus*
**72**	0.000432	0.001098	*Beijerinckia mobilis*
**104**	0.000028	0.000693	*Sphingopyxis witflariensis*
**113**	0	0.000663	*Stella humosa*
**115**	0	0.000622	*Loktanella salsilacus*
**108**	0.000051	0.000647	*Novosphingobium panipatense*
**26**	0	0.000559	*Acinetobacter johnsonii*
**48**	0	0.00052	*Flavobacterium psychrophilum*
**166**	0	0.000508	*Rhodococcus fascians*
**65**	0.00064	0.001148	*Methylobacterium organophilum*
**103**	0.000215	0.000655	*Sphingomonas ursincola*
**144**	0.00007	0.000462	*Acinetobacter lwoffii*
**191**	0.00006	0.000406	*Beijerinckia derxii subsp. Venezuelae*
**19**	0.000154	0.000498	*Brevundimonas variabilis*
**178**	0.000043	0.000378	*Pseudorhodoferax soli*
**23**	0.000017	0.000348	*Flavobacterium psychrophilum*
**192**	0	0.000328	*Methylovirgula ligni*
**49**	0.000058	0.000354	*Flavobacterium aquatile*
**8**	0	0.00029	*Methylosinus sporium*
**9**	0.000062	0.000245	*Sphingomonas wittichii*
**169**	0.000207	0.000287	*Corynebacterium tuberculostearicum*
**111**	0.000416	0.000202	*Mycoplana bullata*
**7**	0.000343	0	*Singularimonas variicoloris*
**175**	0.000348	0	*Simplicispira limi*
**13**	0.000409	0	*Chelatococcus daeguensis*
**16**	0.000441	0	*Sphingomonas wittichii*
**150**	0.000678	0.000214	*Sphingomonas wittichii*
**75**	0.000558	0	*Pseudochrobactrum kiredjianiae*
**45**	0.000606	0.000032	*Chryseobacterium indologenes*
**173**	0.000661	0.000034	*Methylobacterium rhodesianum*
**30**	0.002932	0.001072	*Methylobacterium populi*
**62**	0.004156	0.001574	*Methylobacterium salsuginis*
**149**	0.007494	0.00191	*Methylobacterium rhodesianum*
**50**	0.889343	0.227483	*Methylobacterium rhodesianum*

Differentially abundancy were calculated by using Metastats (using a FDR correction, p<0.01).

**Table 5:** Detailed information related to the OTU differentially abundant between individuals belonging to group 2 and group 3.

N° OTU	mean abundance in group 2	mean abundance in group 3	Best hit on Blast
**63**	0.002374	0.027857	*Paracoccus haeundaensis*
**55**	0	0.008105	*Bradyrhizobium pachyrhizi*
**54**	0.000782	0.007992	*Methylopila capsulata*
**134**	0.000155	0.00635	*Acinetobacter lwoffii*
**129**	0	0.005644	*Pseudomonas hibiscicola*
**143**	0.000513	0.005914	*Dokdonella koreensis*
**124**	0.000109	0.004908	*Nevskia soli*
**40**	0.000306	0.004576	*Flavobacterium aquatile*
**123**	0.000266	0.004124	*Acinetobacter haemolyticus*
**71**	0.00054	0.004232	*Sphingomonas faeni*
**38**	0.001334	0.004547	*Sejongia jeonii*
**156**	0.000306	0.003487	*Sphingopyxis alaskensis*
**39**	0.000144	0.00318	*Flavobacterium psychrophilum*
**78**	0.000761	0.003657	*Rhodobacter capsulatus*
**137**	0.000339	0.002615	*Acinetobacter johnsonii*
**47**	0.000087	0.002082	*Flavobacterium psychrophilum*
**164**	0.000236	0.002164	*Rhodococcus fascians*
**4**	0	0.00161	*Methylosinus sporium*
**182**	0.000579	0.002172	*Flavobacterium aquatile*
**3**	0	0.001358	*Rhodobacter changlensis*
**184**	0	0.001156	*Flavobacterium aquatile*
**42**	0.000252	0.001071	*Flavobacterium psychrophilum*
**12**	0.001029	0	*Pseudorhodoferax soli*
**6**	0.001033	0	*Methylovirgula ligni*
**27**	0.001814	0.000082	*Sphingopyxis chilensis*
**50**	0.677999	0.227483	*Methylobacterium rhodesianum*

Differentially abundancy were calculated by using Metastats (using a FDR correction, p<0.01).

**Figure 5:**
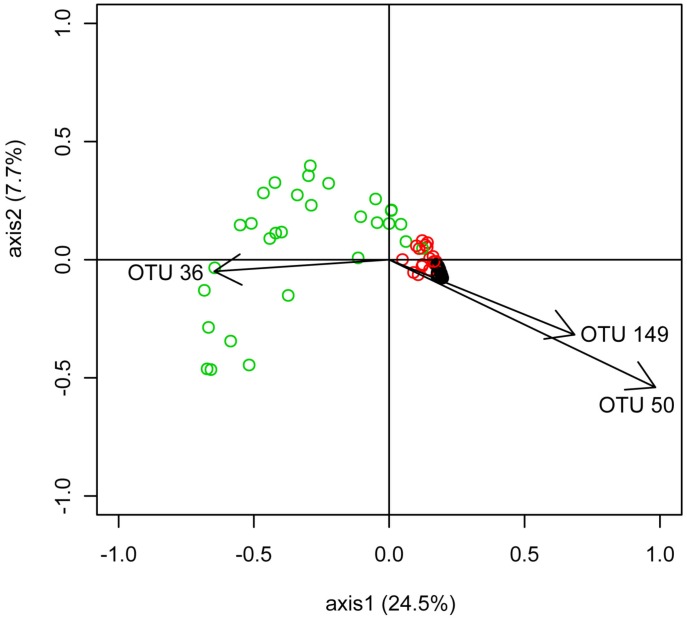
PCoA analysis of the microbiome for all 86 F_2_ individuals based on the Yue & Clayton measure of dissimilarity (Thetayc). Black circles represent individuals belonging to the group 1, red circles represent individuals belonging to the group 2 and green circles represent individuals belonging to the group 3. Arrows represent genus, which are significantly correlated with the axis. The first and second axes represented 24.5% and 7.7% of the variation respectively. The R-squared between the original distance matrix and the distance between the points in 2D PCoA space was 0.87.

**Figure 6:**
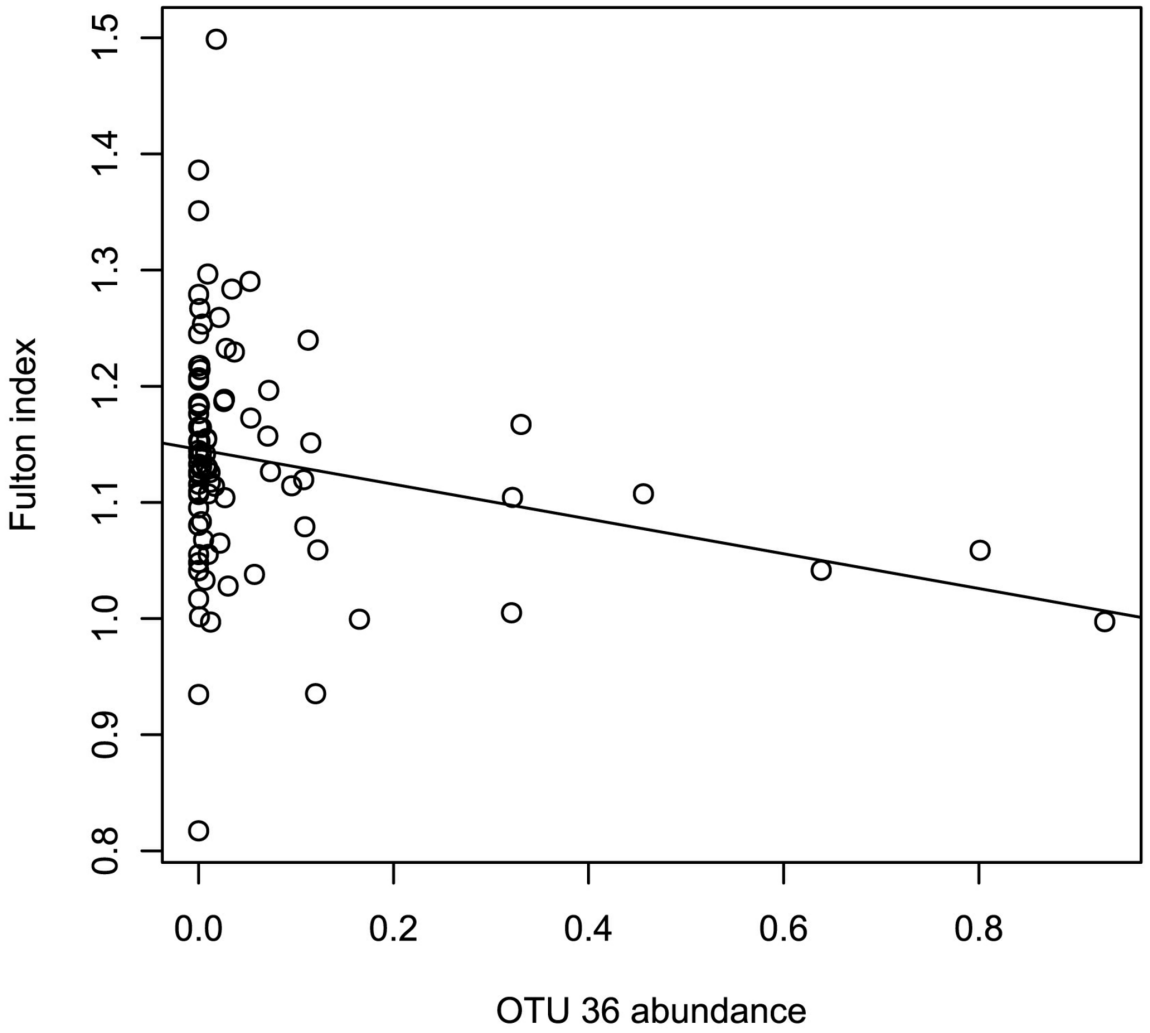
Relationship between the Fulton Index and and the abundance of the OTU 36. A linear regression is observed based on a linear model (Shapiro test for Normality: p-value  = 0.07, Linear model: p-value = 0.02741, F = 5.0388, Rsq = 0.04536).

PCoA analysis displayed the same patterns of divergence between the three groups (Figure 5). Each group found in the Unifrac analysis was highlighted in the PCoA. To understand which OTUs were responsible for the differentiation of the cluster on the PCoA axes 1 and 2, individual correlation coefficients were calculated. Three OTUs were highly correlated with the two axes (σ>0.6); OTU 36, 50 and 149. Those OTU belonged to two species: *Flavobacterium psychrophilum* (OTU 36) and *M. Rhodesianum* (OTU 50 and 149). The negative correlation between *Methylobacterium* and *Flavobacterium* was further confirmed by the co-occurrence analysis, which identified potential taxonomic interactions between genera. Two significant correlations between genera were detected: a positive correlation between *Maritimibacter* and *Micrococcus* (σ = 0.69, p-value <0.01) and a negative correlation between *Methylobacterium* and *Flavobacterium* (σ = −0.63, p-value <0.01). Furthermore, variance analysis of OTU 36′ abundance showed that it was influenced by the Fulton index (Shapiro test for Normality: p-value  = 0.07, Linear model: p-value = 0.02741, F = 5.0388, Rsq = −0.1496). A negative relationship between Fulton index and the OTU 36 was observed (Figure 6).

In the F2 fish progeny, three significant QTLs (at the genome-wide level) were identified on two linkage groups (LG 11 and LG 16). One major QTL per strain was detected respectively for *Lysobacter*, *Rheinheimera* and *Methylobacterium* counts (LOD score  = 9.89, 7.46 and 3.48 respectively). For each of these traits, the total PVE (percent variance explained) of the QTL were estimated to 17.01%, 31.05% and 41.1% for *Methylobacterium*, *Rheinheimera* and *Lysobacter* respectively. The most probable positions of these QTL, their respective 95% CIs, the closest linked molecular markers (one per QTL) as well as additive and dominance effects are presented in Table 6.

**Table 6:** Descriptive statistics, including the LOD score, the position, 95% CI, PVE (%), the associated P-value of each QTL linked to every phenotype related to bacteria counts trait (LOD, Log_10_ of the odd ratio; 95% CI, 95% confidence interval; PVE, percent variance explained) [23].

Phenotype	Linkage Group	Pos (cM)	95% CI (cM)	LOD	*p* value (F)	PVE (in %)	Nearest marker
***Lysobacter***	11	62	56.2–68.2	9.89	0.000	41.1	sf003455
***Rheinheimera***	16	42.8	39.3–45.5	7.46	0.000	31.05	SFO-D91
***Methylobacterium***	16	39	34.5–43.5	3.48	0.001	17.02	SFO-D91

## Discussion

Inter-individual variations in host microbiota has been well documented (e.g. [1]. Such variations occur even in hosts with identical genetic background, as observed in monozygotic twins [3] indicating that both genetic and environmental conditions play a role in the modulation of host microbiota. The gold standard forward genetics technique to identify areas of the genome that relate to certain phenotypes is to make a cross between genetically divergent individuals [32]. In this study, we focused on the structural variation of the skin microbiota (i.e abundance of each bacterial genus) of an F2 intercross originally generated from two genetically contrasted strains of brook charr. To our knowledge, this is the first study that identified host genomic regions associated with the abundance of specific microbiota strains in a non-model vertebrate.

The deep taxonomic analysis of the fish skin microbiota indicates that two phyla dominate: *Proteobacteria* (88.7%), followed by *Bacteroidetes* (9.7%). Those two phyla are mainly represented by a single OTU each; 50 and 36 respectively. OTU 50 is classified as *M. rhodesianum*. OTU 36 is classified as *F. psychrophilum*, the causative agent of the cold water disease, a pervasive infectious disease in farmed fish [33], [34]. This disease especially affects salmonids at early life-stages, and it is well documented that both stressful conditions, and injuries facilitate infection triggering [33], [35]. According to the dendrogram (Figure 2), each individual's bacterial communities clustered into three groups. Similar clustering was also found with the second normalization method (subsampling of the same number of sequences for each samples). The same result was obtained with PCoA analysis and furthermore the Pearson correlation indicates that the genus *Flavobacterium* and *Methylobacterium* are negatively correlated. In the PCoA, two species (*F. psychrophilum* and *M. rhodesianum*) are correlated in opposite ways with the axis 1 which discriminate the three groups. We assume that two or more species which seem to be mutually exclusive are involved in a negative relationship [36]. All together those results indicate that the antagonistic relationship between those two dominant species is very likely playing a key role in shaping the structure of the individual's microbiota.

Because all fish progenies were reared in the same tank, and under the same environmental conditions since birth, environment had likely little influence on the phenotypic variation observed in this study. Yet, the individual's microbiota either clustered in one of two closely related groups, or formed divergent outliers. Sex-specific variations had little influence on individual clustering since both males and females were present in each defined group and there was no correlation between sex and groups. The difference between male and female is based on 22 OTUs which are less abundant than 0.1% of the community and are then classified as part of the rare biosphere. Previous studies observed similar individual variations of the microbiota in human or mouse [1], [2], [32].

Three OTUs classified into two species were responsible for the inter-individual differentiation; *M. rhodesianum and F. psychrophilum*. Interestingly, *Flavobacterium* and *Methylobacterium*, which are the most abundant genera, negatively co-occurred in the samples, indicating that the relationship between those two genera is based on competition. Furthermore, a strong negative correlation was found between the OTU 36 and 50, thus supporting a strong antagonistic relationship between the two species, *F. psychrophilum* and *M. rhodesianum*.

*M. rhodesianum* produces poly-β-hydroxybutyrate, a polymer of short-chain fatty acid, known to inhibit the growth of pathogens like enterobacteria and *Vibrio sp*. [37], [38], [39], [40], [41], [42]. Taken together, this suggests that a reduction of *M. rhodesianum* abundance allows colonization or over-growth by other bacteria, some of which are pathogenic.

Evidently, a change in skin microbiota taxonomic structure favors opportunist bacteria and especially opportunistic pathogens like *F. psychrophilum, Acinetobacter haemolyticus, Acinetobacter johnsonii and Acinetobacter junii*, which have a higher abundance in group 2 compared with group 1 Such a disturbance in the microbiome homeostasis is called dysbiosis, and its occurrence enlightens the importance of the function of *M. rhodesianum* in controlling the balance between both other commensal bacteria and opportunistic pathogens. Furthermore, *Flavobacterium psychrophilum* was negatively correlated to both *M. rhodesianum* abundance and Fulton index. The Fulton index is a condition factor used as a proxy for fish health status. Therefore, it suggests that skin microbiota from the weakest host individuals were unable to prevent colonization by the opportunistic pathogen *F. psychrophilum*. Furthermore, skin microbiota taxonomic structure varies significantly across the F2 progeny. This result added to the fact that *i* the genetics of the host is the only variable in the experiment, and *ii* the finding of three QTLs associated to the abundance of bacterial genera with high PVE, suggest that host genotype influences the abundance of commensal strains e.g. *Methylobacterium*, which will regulate the abundance of *F. psychrophilum*.

We found three QTL associated with the abundance of three genera: *Lysobacter*, *Rheinheimera* and *Methylobacterium*, all of which were observed to provide antimicrobial compounds [39], [40], [41], [42], [43], [44], [45], [46], [47]. These finding strongly suggests that host genotype influences abundance of specific commensal strains, and possibly targets their recruitment. The most compelling evidence concerns the QTL associated with *Methylobacterium* (PVE = 17.01%): as previously described above, *Methylobacterium* is influential on the structure and the homeostasis of the microbiota. Its abundance is inversely correlated with those of the pathogen *F. psychrophilum*. The targeted recruitment of *M. rhodesianum* mediated by the host genotype is therefore a strategy to prevent pathogen growth *via* harnessing antagonistic relationship towards resources use [48]. To our knowledge, this is only the second study that identified QTL associated with microbial variation among individuals. A study on murine gut microbiota showed similar results, in which McKnite *et al.* (2012) found 5 QTL located on four chromosomes influencing the variation of different taxa. The variance explained by their QTL is in the same range of our (20% to 27% of the variance explained for McKnite *et al.* (2012)). We also found QTLs with a major effect on the variance of genus abundances (PVE ranging for 17.02 to 41.1%). Linkage analysis on genus abundance data strongly evidenced the influence of host genetics on the modulation and/or recruitment for those genera. Therefore, brook charr immunity involves both specific commensal strains recruitment and nuclear gene expression, as previously evidenced in [49], those are under the control of the host genotype.

The study of the genetic architecture underlying the regulation of bacterial abundance further highlights the coevolutionary pattern between host, commensals, and pathogens. However, identifying genes located in the genomic regions (QTL) linked to the abundance of microbial partners will be far more challenging as the genome of brook charr is currently not fully sequenced and poorly annotated compared to the mouse genome. The markers surrounding the QTL regions may define regions to be deeply sequenced in future work to identify potential underlying genes and their associated functions. Evidently, those markers will be invaluable to conduct highly innovative genetic breeding programs targeting disease resistant host strains via the recruitment of highly resilient microbiota.

## Supporting Information

Figure S1 **Rarefaction curves for each group defined by the dendrogram analysis based upon ThetaYC index (see** **figure 2** **).** Each group curve reaches a plateau thus indicating the depth of sequencing is sufficient.
(EPS)

Figure S2 **Alpha diversity of each group normalized by subsampling of the same number of reads per sample.** Each group was defined by the dendrogram analysis based upon ThetaYC index performed on the normalized dataset. Alpha-diversity was calculated by the non-parametric index of Shannon. Statistical differences (represented by the asterisk) were calculated with a wilcoxon test with a correction for multiple testing (Holm, p-values <1.10^−4^).
(EPS)

Figure S3 **PCoA analysis of the microbiome for all 86 F_2_ individuals.** This PCoA was performed on a normalized dataset with the second method of normalization (subsampling of the same number of reads per sample). Black circles represent individuals belonging to the group 1, red circles represent individuals belonging to the group 2 and green circles represent individuals belonging to the group 3. Arrows represent genera, which are significantly correlated with the axis. The first and second axes represented 24.5% and 7.8% of the variation respectively. The R-squared between the original distance matrix and the distance between the points in 2D PCoA space was 0.88.
(EPS)

Table S1 **Differentiation between the three groups of OTU calculated with an analysis of similarity (ANOSIM, statistic** ***R*** **).** The q-values represent p-values corrected with Bonferroni and considered as significant when p<0.05. Note: The analysis was performed on a dataset normalized by two methods: zscore and subsampling. Both analyses gave the same results, each group being significantly different from the two others.
(DOC)
