# Supplementary material for: Inter Individual Variations of the Fish Skin Microbiota: Host Genetics Basis of Mutualism?
Source: PLoS One. 2014 Jul 28;9(7):e102649. doi: 10.1371/journal.pone.0102649 (PMC4113282; doi:10.1371/journal.pone.0102649)
Supplement: Table S1 — Differentiation between the three groups of OTU calculated with an analysis of similarity (ANOSIM, statistic R ). The q-values represent p-values corrected with Bonferroni and considered as significant when p<0.05. Note: The analysis was performed on a dataset normalized by two methods: zscore and subsampling. Both analyses gave the same results, each group being significantly different from the two others. (DOC) [file pone.0102649.s004.doc]

Table S1. Differentiation between the three groups of OTU calculated with an analysis of similarity (ANOSIM, *R* statistic). The analysis was performed on a dataset normalized by two methods: zscore and subsampling. Both analyses gave the same results, each group being significantly different from the two others. The q-values represent p-values corrected with Bonferroni and are considered as significant when p < 0.05.

| **Anosim(sub-sampling)** | **R-value** | **q-value** |
| --- | --- | --- |
| Group1-group2-group3 | 0.49253 | <0.001 |
| group1-group3 | 0.757003 | <0.001 |
| group1-group2 | 0.588731 | <0.001 |
| group2-group3 | 0.117265 | 0.034 |
| **Anosim(z-score)** | **R-value** | **q-value** |
| Group1-group2-group3 | 0.49215 | <0.001 |
| group1-group3 | 0.742955 | <0.001 |
| group1-group2 | 0.593233 | <0.001 |
| group2-group3 | 0.120874 | 0.028 |
